# Comparative Analysis of Growth and Photosynthetic Characteristics of (*Populus simonii* × *P*. *nigra*) × (*P*. *nigra* × *P*. *simonii*) Hybrid Clones of Different Ploidides

**DOI:** 10.1371/journal.pone.0119259

**Published:** 2015-04-13

**Authors:** Xiyang Zhao, Ying Li, Mi Zheng, Xiuyan Bian, Mengran Liu, Yanshuang Sun, Jing Jiang, Fuwei Wang, Shuchun Li, Yonghong Cui, Guifeng Liu, Chuanping Yang

**Affiliations:** 1 State Key Laboratory of Tree Genetics and Breeding (Northeast Forestry University), Northeast Forestry University, Harbin, 150040, China; 2 Tree Seedling Management Station, Forestry Department of Jilin Province, Changchun, 130000, China; 3 Seed Orchard of Siping, Siping, 136000, China; Pennsylvania State University, UNITED STATES

## Abstract

To evaluate differences among poplar clones of various ploidies, 12 hybrid poplar clones (*P*. *simonii* × *P*. *nigra*) × (*P*. *nigra* × *P*. *simonii*) with different ploidies were used to study phenotypic variation in growth traits and photosynthetic characteristics. Analysis of variance showed remarkable differences for each of the investigated traits among these clones (*P* < 0.01). Coefficients of phenotypic variation (PCV) ranged from 2.38% to 56.71%, and repeatability ranged from 0.656 to 0.987. The Pn (photosynthetic rate) photosynthetic photon flux density (PPFD) curves of the 12 clones were S-shaped, but the Pn-ambient CO_2_ (Ca) curves were shaped like an inverted “V”. The stomatal conductance (Gs)-PPFD and transpiration rate (Tr)-PPFD curves had an upward tendency; however, with increasing PFFD, the intercellular CO_2_ concentration (Ci)-PPFD curves had a downward tendency in all of the clones. The Pn-PPFD and Pn-Ca curves followed the pattern of a quadratic equation. The average light saturation point and light compensation point of the triploid clones were the highest and lowest, respectively, among the three types of clones. For Pn-Ca curves, diploid clones had a higher average CO_2_ saturation point and average CO_2_ compensation point compared with triploid and tetraploid clones. Correlation analyses indicated that all investigated traits were strongly correlated with each other. In future studies, molecular methods should be used to analyze poplar clones of different ploidies to improve our understanding of the growth and development mechanisms of polyploidy.

## Introduction

Poplars (*Populus* spp.) are some of the most important economic tree species in the temperate regions of the world [1, 2]. With the publication of the *P*. *trichocarpa* genome [3], poplar has become a model organism for the study of trees and is the most intensively studied tree genus. In 1959, the Chinese Academy of Forestry (CAF) conducted a large-scale crossing experiment on poplar. After 20 years, hybridized combinations of *P*. *simonii* × *P*. *nigra* and *P*. *nigra* × *P*. *simonii* were selected as excellent materials for afforestation in Northern China because of the rapid growth, excellent wood properties, high cold resistance and drought endurance of their offspring [4, 5]. In recent years, many studies have been conducted using these two families as materials, which have mainly focused on growth traits [6, 7], resistance [8], physiology [9], and molecular research [10, 11].

Polyploidy is a ubiquitous phenomenon in higher plants. It is estimated that polyploidy has occurred in 50–70% of flowering species [12, 13], most of which have experienced one or more polyploidization events during their evolution [14]. The increase in chromosomes has resulted in increased gene dosages and cell volumes [15]. Therefore, polyploid plants usually have larger leaves, greater height and diameter, and an increased ability to adapt to their environment [16, 17]. The first natural European aspen polyploid (*Populus tremula*) was discovered by Nilsson-Ehle [18] in Sweden. Since then, breeders have paid close attention to different ploidy level (diploid, triploid and tetraploid) breeding in forest trees because of the huge growth of forestry. Many natural triploid poplars were found in the Soviet Union, Sweden, Finland and other countries [19–22]. In research on different types of polyploids, triploid poplars are often characterized as having fast growth, large leaves, vigor and low fertility compared with their diploid counterparts [23]. However, fewer comparative studies on poplars with different ploidies, including tetraploids, have been reported [24]. In this study, with the aim of obtaining high growth rate and highly resistant offspring, *P*. *simonii* × *P*. *nigra* and *P*. *nigra* × *P*. *simonii* were selected as parents. Colchicine was used in the crossing experiment and many offspring were obtained with different ploidies. Twelve offspring with different ploidies were used as materials in this experiment. Our primary objectives were to explore the variation in growth, photosynthesis and chlorophyll fluorescence traits among hybrid clones with different ploidies, and to provide a theoretical basis for polyploid poplar clone selection.

## Materials and Methods

### Plant materials

**Fig 1:**
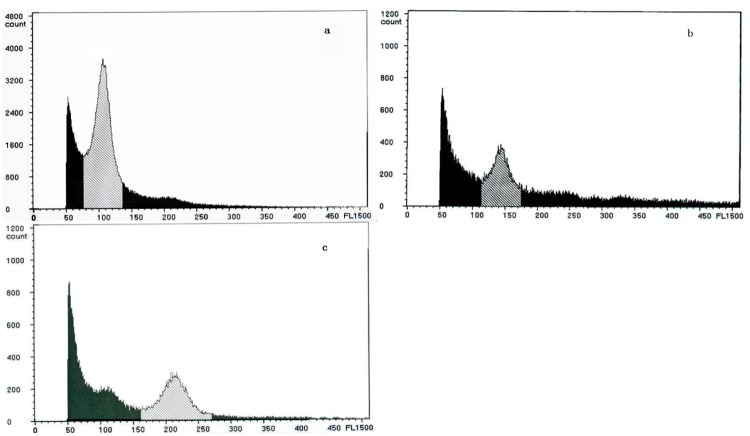
DNA content of sn2.1 (a: the main peak at channel 100), sn3.8 (b: the main peak at channel 150) and sn4.1 (c: the main peak at channel 200). The ordinate (count) represents the relative value of the cell population, the abscissa (FL1) represents the passageway value of fluorescence. The abscissa location which peak point corresponding were the ploidy of test sample.

Female *Populus simonii* × *P*. *nigra* and male *P*. *nigra* × *P*. *simonii* plants (both of the parents were diploids) were selected as parents, and artificially controlled pollination was performed in 2006. During male and female flower development, a 0.5% colchicine solution was injected into flower buds at the beginning of reduction mitosis to obtain reduplicated pollen and metrocytes. The crossing experiment was carried with reduplicated pollen and reduplicated metrocytes to obtain polar seeds with different ploidies. After sowing, the DNA contents in the leaves were evaluated by flow cytometry using the method of Zhang [25]. Twelve hybrid clones with different ploidies [(*Populus simonii* × *P*. *nigra*) × (*P*. *nigra* × *P*. *simonii*)] were used for this study, including three tetraploid clones (sn4.1, sn4.2 and sn4.3), five triploid clones (sn3.1, sn3.2, sn3.4, sn3.8 and sn3.9) and four diploid clones (sn, sn2.1, sn2.2, sn2.3). The total DNA contents of each clone were measured and the percentages of the cells that showed varied DNA content were processed by the DPAC software. The results of sn2.1, sn3.8 and sn4.1 are shown in Fig 1. In 2013, 50 hardwood cuttings (15 cm long each) of each clone were collected from 1-year-old seedlings in a greenhouse at the Northeast Forestry University. All cuttings were moistened and stored in plastic bags at 4°C for about 2 weeks and then planted in 1.5-L plastic pots containing garden soil mixed with peat at a ratio of 3:1 (v/v). The cuttings were grown in artificial climate chambers of Northeast Forestry University under a cycle of 1000 μmol m^-2^ s^-1^ light for 16 h from 8:00 to 24:00 and dark for 8 h. The temperature and humidity were set at 27°C and 60%, respectively.

### Growth traits observation

After growth for 3 months, 30 healthy and uniform cuttings of each clone were used as materials to measure tree height (H), basal diameter (BD) and leaf number (LN) of each plant. Nine leaves (4–12) from a shoot tip per cutting were taken to measure the leaf area (LA). Each leaf and stem (the whole stem) was weighed for the leaf fresh weight (LFW) and stem fresh weight (SFW). Leaves and stems were then dried in an oven (90°C) to a constant weight and measured for leaf dry weight (LDW) and stem dry weight (SDW).

### Photosynthetic analysis

Nine uniform plants of each clone were selected to measure the photosynthetic parameters. Net photosynthesis rate (Pn), intercellular CO_2_ concentration (Ci), stomatal conductance (Gs) and transpiration rate (Tr) were measured from 8:30 a.m. to 11:30 a.m. using a Lico-6400 portable photosynthesis measuring system (Li-Cor Inc., Lincoln, NE, USA) on the third to fifth fully expanded leaf of each plant. The conditions for photosynthetic trait measurements were: leaf temperature 28°C, photosynthetic photon flux density (PPFD) 1400 μmol m^-2^ s^-1^, relative air humidity (RH) 60% and ambient CO_2_ concentration (Ca) 400 μmol mol^-1^.

### Light and CO_2_ response measurements

Pn, Gs, Ci and Tr responses to PPFD and Ca were also measured. Photosynthesis traits-PPFD curves were produced under a Ca concentration of 400 μmol mol^-1^, leaf temperature around 28°C and relative humidity about 60%. The light intensities varied with 0, 20, 50, 80, 100, 200, 400, 600, 800, 1000, 1200, 1400, 1600, 1800 and 2000 μmol m^-2^ s^-1^. Response curves were modeled by a quadratic equation (Eq 1) according to Zhao [26, 27]. Light saturation point (LSP) and light compensation point (LCP) were evaluated by fitting data to the model function. Pn-Ca curves were measured under saturating light intensity (1400 μmol m^-2^ s^-1^), and a temperature and humidity around 28°C and 60%. The CO_2_ concentrations in the leaf chamber were set at 0, 50, 80, 100, 200, 300, 400, 600, 800, 1000, 1200, 1400 and 1600 μmol mol^-1^. Model 1 was used to create response curves and to evaluate the carbon dioxide saturation point (CSP) and carbon dioxide compensation point (CCP).

$$Y=b_{0}+b_{1}X+b_{2}X^{2}$$ (1)

Where *Y* was the Pn value, *X* was the PPFD (Ca), *b*_0_ was constant, and *b*_1_ and *b*_2_ were coefficients, respectively.

### Chlorophyll fluorescence parameter measurements

Chlorophyll fluorescence parameters were measured using a pulse amplitude modulation chlorophyll fluorometer MINI-PAM2500 (Walz, Effeltrich, Germany). Minimal fluorescence, F_0_, was measured in 30-min dark-adapted leaves using weak modulated light of < 0.15 μmol m^-2^ s^-1^. Maximal fluorescence, Fm, was measured after a 0.8-s saturating white light pulse (6000 μmol m^-2^ s^-1^) in the same leaves. Maximal variable fluorescence (F_v_ = F_m_–F_0_) and the photochemical efficiency of PSII (Fv/Fm) for dark-adapted leaves were calculated.

### Data analysis

Statistical analyses were carried out using the Statistical Product and Service Solutions (SPSS 19.0) software. All the growth traits, instantaneous photosynthetic and chlorophyll fluorescence parameters were compared using analysis of variance; the significance of fixed effects was tested with *F*-tests.

The coefficient of phenotypic variation (PCV) of all the investigated traits was calculated using the following formula [28]:

$$PCV=\frac{SD\times 100}{\overset{-}{X}}$$ (2)

where $\bar{X}$ is the phenotypic mean of the trait and SD is the standard deviation of the trait.

The repeatability (*R*) of all the investigated traits was calculated as follows [29]:

$$R=\frac{\sigma_{c}^{2}}{\sigma_{c}^{2}+\sigma_{e}^{2}}$$ (3)

where $\sigma_{c}^{2}$ is the genetic variance component between clones and $\sigma_{e}^{2}$ is the error variance component.

The phenotype correlation r_A_(xy) of traits x and y was calculated as follows [30]:

$$r_{A}(xy)=\frac{\sigma_{a(xy)}}{\sqrt{\sigma_{a(x)}^{2}\cdot \sigma_{a(y)}^{2}}}$$ (4)

where $\sigma_{a(x)}^{2}$ is the clone variance component for the trait x, $\sigma_{a(y)}^{2}$ is the clone variance component for the trait y and σ _a (xy)_ is the clone covariance component.

## Results

### Variation among growth traits

The results of ANOVA for all growth traits are presented in Table 1. There were significant differences among the clones with different ploidies (*P* < 0.01) and clones, except for LFW among clones (*P* = 0.014) based on overall *F*-tests. The PCVs of different growth traits ranged from 12.60% to 43.36%, with the value of BD being the lowest and that of SFW being the highest, respectively. The repeatabilities (*R*) of the different traits are also presented in Table 1. The R values for all growth traits were higher than 0.60. High R and PCV are favorable for excellent clone selection.

**Table 1:** ANOVA analyses, PCV and R of different traits among different ploidies and clones.

Traits	Variation source	*SS*	*df*	*MS*	*F*	*P* value	*PCV* (%)	*R*
H	Ploidies	1058.400	2	529.200	41.422	0.000**		
	Clones	1197.333	11	108.848	9.242	0.000**	18.76	0.892
BD	Ploidies	2.896	2	1.448	12.221	0.000**		
	Clones	4.058	11	0.369	3.221	0.008**	12.60	0.690
LN	Ploidies	179.706	2	89.853	14.886	0.000**		
	Clones	272.889	11	24.808	5.617	0.000**	18.86	0.822
LA	Ploidies	1365.937	2	682.969	17.530	0.000**		
	Clones	1867.071	11	169.734	5.192	0.000**	27.89	0.807
SFW	Ploidies	77.325	2	38.663	31.385	0.000**		
	Clones	104.961	11	9.542	17.593	0.000**	43.36	0.943
SDW	Ploidies	3.054	2	1.527	10.839	0.000**		
	Clones	6.310	11	0.574	9.878	0.000**	42.08	0.899
LFW	Ploidies	.568	2	.284	11.716	0.000**		
	Clones	0.782	11	0.071	2.907	0.014*	22.73	0.656
LDW	Ploidies	.079	2	.040	15.886	0.000**		
	Clones	0.101	11	0.009	3.613	0.004**	27.01	0.723

**(*) indicated variance is significant at the 0.01(0.05) level

### Average growth traits for different ploidy poplar clones

The average growth traits of hybrid poplar clones with different ploidies are summarized in Table 2. The overall average tree height and average base diameter were 35.54 cm and 3.49 mm, respectively, for all plants. The average values of all growth traits in the triploids were higher than those in the tetraploid and diploid hybrid clones, and the diploid hybrid clones had the lowest growth traits.

**Table 2:** Average growth traits for different hybrid clones of different ploidies.

Ploidy	clone	H (m)	BD (mm)	LN	LA (cm^2^)	SFW (g)	SDW (g)	LFW (g)	LDW (g)
Tetraploid	4.1	33.00±2.65	3.51±0.18	15.67±2.08	27.44±1.64	3.88±0.16	0.95±0.18	0.78±0.16	0.21±0.04
	4.2	35.00±2.68	3.54±0.24	16.67±2.08	30.00±1.73	3.92±0.80	0.99±0.14	0.88±0.19	0.27±0.06
	4.3	31.00±2.44	3.52±0.12	15.67±2.08	28.11±2.03	3.85±0.46	0.90±0.19	0.79±0.10	0.21±0.05
	average	33.00±2.87	3.52±0.16	16.00±1.87	28.52±2.61	3.88±0.47	0.95±0.19	0.82±0.14	0.23±0.05
Triploid	3.1	39.33±2.08	3.77±0.18	20.67±2.08	37.00±6.09	5.46±0.31	1.46±0.45	0.94±0.12	0.34±0.04
	3.2	41.33±3.15	3.81±0.18	23.33±2.31	39.33±6.37	6.52±0.50	1.61±0.10	1.07±0.20	0.31±0.03
	3.4	38.67±2.03	3.55±0.39	18.00±2.65	31.67±7.64	4.71±0.49	1.03±0.28	0.92±0.20	0.33±0.04
	3.8	45.00±4.58	4.17±0.30	21.33±2.89	47.00±3.67	7.56±0.64	2.21±0.33	1.19±0.11	0.29±0.02
	3.9	39.33±3.21	3.62±0.50	17.00±1.73	36.33±4.73	5.01±0.89	0.96±0.22	0.94±0.10	0.25±0.03
	Average	40.73±3.83	3.78±0.35	20.07±3.10	38.27±8.27	5.85±1.36	1.45±0.53	1.01±0.17	0.31±0.06
Diploid	2.1	30.33±4.93	3.40±0.42	15.67±2.31	27.67±5.49	3.71±0.88	0.87±0.19	0.75±0.23	0.20±0.05
	2.2	25.67±1.15	2.96±0.62	14.00±1.80	23.67±3.79	1.11±0.28	0.61±0.02	0.74±0.20	0.20±0.04
	2.3	28.33±5.03	2.92±0.39	14.67±2.08	19.78±1.58	2.16±0.10	0.85±0.09	0.64±0.10	0.20±0.03
	2.4	29.00±2.65	3.21±0.18	16.67±1.53	26.56±5.87	2.94±0.20	0.94±0.26	0.78±0.07	0.21±0.02
	average	28.33±3.70	3.13±0.42	15.25±1.82	24.42±5.00	2.48±1.08	0.82±0.20	0.73±0.15	0.20±0.04

### Pn-PPFD curves

**Fig 2:**
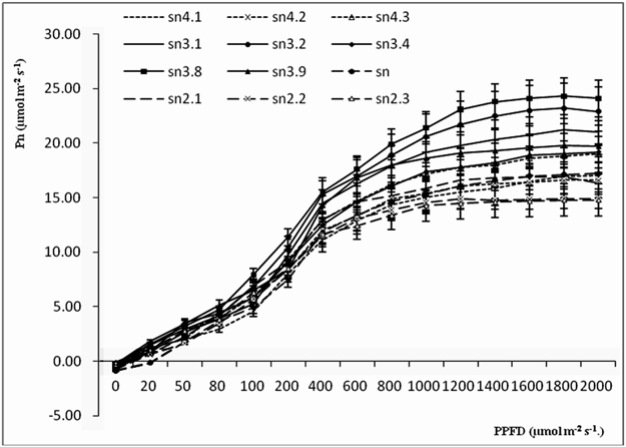
Pn-PPFD curves of poplar clones of different ploidies. Different ploidy clones are shown by different line types and identification. Triploids are shown by solid lines, diploid and tetraploid are shown as different types of dotted and dashed lines.

The Pn-PPFD curves of different poplar clones are shown in Fig 2. The Pn-PPFD curves of all 12 clones were S-shaped. The Pn values of all clones increased as PPFD increased in low light conditions and reached a plateau when PPFD reached 1200–1400 μmol m^-2^ s^-1^. The triploid hybrid clones showed higher Pn values than the diploid and tetraploid hybrid clones. In particular, sn3.8 had the highest Pn values across all light intensities.

### Gs-PPFD, Ci-PPFD and Tr-PPFD curves

**Fig 3:**
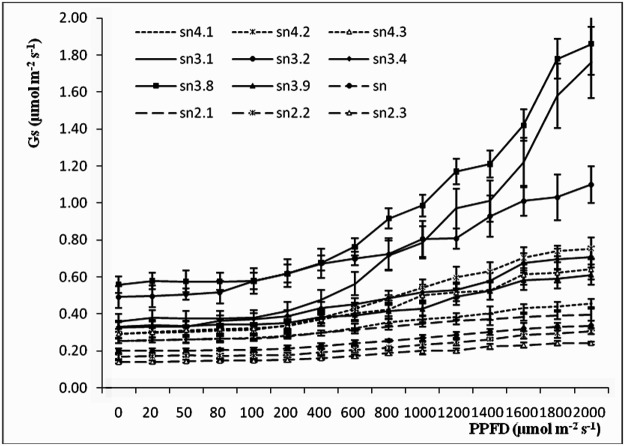
Gs-Par curves of poplar clones of different ploidies.

**Fig 4:**
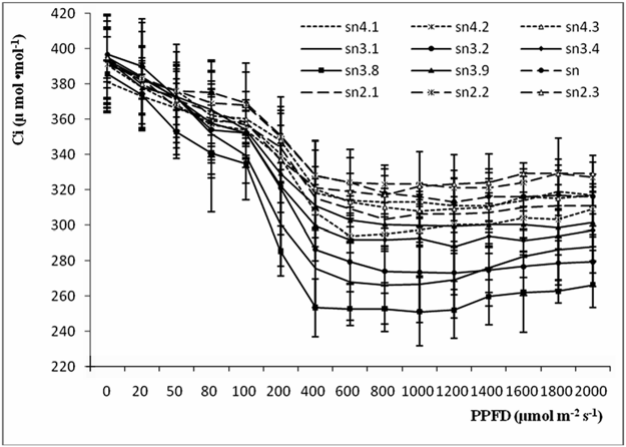
Ci-Par curves of poplar clones of different ploidies.

**Fig 5:**
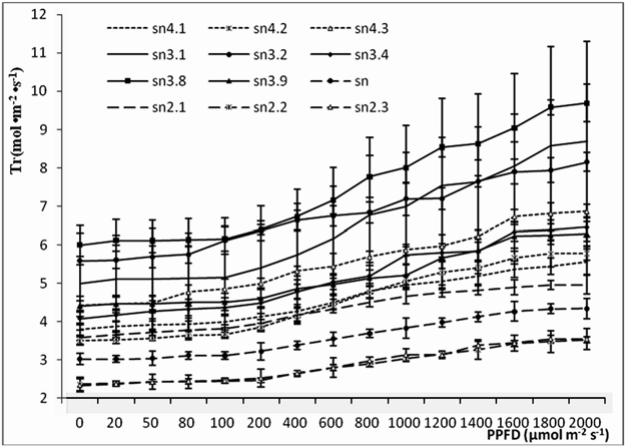
Tr-Par curves of poplar clones of different ploidies.

Gs-PPFD, Ci-PPFD and Tr-PPFD curves are shown in Figs 3–5. Gs and Tr values had an upward tendency in all clones as PPFD increased. Clones sn3.8, sn3.2 and sn3.1 had higher Gs values than the other clones at different light intensities: as PPFD increased, these differences became larger. Clones sn2.3, sn2.2 and sn had obviously lower Gs values than the other clones. By contrast, the Ci-PPFD curves showed a downward tendency, and the Ci values of all clones declined as PPFD increased. This indicated that as Pn values increased, more CO_2_ was needed for photosynthesis, and the concentration of CO_2_ used between the cell spaces decreased. In the Ci-PPFD curves, the Ci values of sn2.3, sn2.2 and sn were higher than those of the other clones, while the Ci values of sn3.8, sn3.2 and sn3.1 were much lower.

### Pn-PPFD curve model

**Fig 6:**
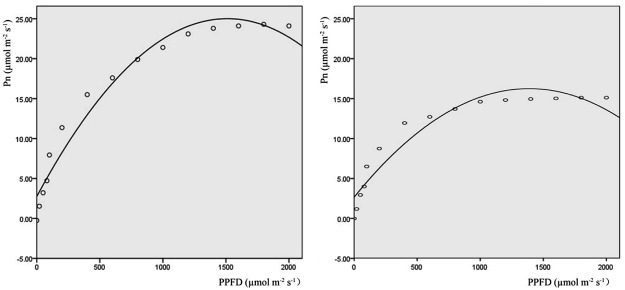
Clone sn3.8 (left) and sn2.3 (right) Pn-PPFD simulated curve (solid lines are simulated curves and white circles are the observed data).

To understand the change in Pn values at different levels of PPFD, quadratic functions were used to simulate the Pn-PPFD relationship. Two simulation curves for sn3.8 and sn2.3 are presented in Fig 6. The parameters used for the simulation curves are listed in Table 3. Parameter *R* ^*2*^ was higher than 0.9, which indicated that the model was effective. The parameters used for LSP and point LCP are also presented in Table 3. The average LSP of the triploid poplar clones exceeded that of the tetraploid and diploid clones. The LCP of the triploid poplar clones was lower than that of the diploid and tetraploid clones, which suggested that the triploid poplar clones had greater adaptability to different light intensities. When the PPFD reached the LCP, different clones expressed Pn differently. Pn varied from 15.90 μmol m^-2^ s^-1^ (sn2.3) to 25.01 μmol m^-2^ s^-1^ (sn3.8) and the average Pn of the triploid clones was higher than that of the tetraploids and diploids.

**Table 3:** Parameters for each Pn-PPFD simulated curve.

Ploidy	clone	a	b	c	LSP	LCP	Pn	*R* ^*2*^
Tetraploid	sn4.1	-7.007E-06	0.0210	1.710	1496.91	7.07	17.41	0.955
	sn4.2	-7.452E-06	0.0213	2.354	1428.14	5.17	17.55	0.947
	sn4.3	-8.096E-06	0.0237	2.244	1465.79	4.86	19.64	0.951
	average				1463.61	5.70	18.20	
Triploid	sn3.1	-9.301E-06	0.0273	1.930	1467.55	8.74	21.96	0.960
	sn3.2	-9.488E-06	0.0285	2.406	1502.98	3.85	23.84	0.966
	sn3.4	-7.815E-06	0.0233	2.383	1489.67	5.67	19.72	0.957
	sn3.8	-9.745E-06	0.0295	2.744	1511.49	2.91	25.01	0.965
	sn3.9	-9.193E-06	0.0260	2.603	1415.46	3.06	21.02	0.956
	average				1477.43	4.84	22.31	
Diploid	sn	-7.681E-06	0.0225	1.420	1465.54	23.96	17.92	0.953
	sn2.1	-8.445E-06	0.0232	2.290	1371.51	10.05	18.17	0.926
	sn2.2	-7.681E-06	0.0210	1.966	1365.92	13.22	16.30	0.929
	sn2.3	-6.923E-06	0.0192	2.556	1388.24	10.71	15.90	0.922
	average				1397.80	14.48	17.07	

Parameters a, b and c are coefficients of the quadratic function. *R* ^*2*^ is the criterion for evaluating the effectiveness of the model. The units for LSP, LCP and Pn are μmol m^-2^ s^-1^.

### Pn-Ca curves

**Fig 7:**
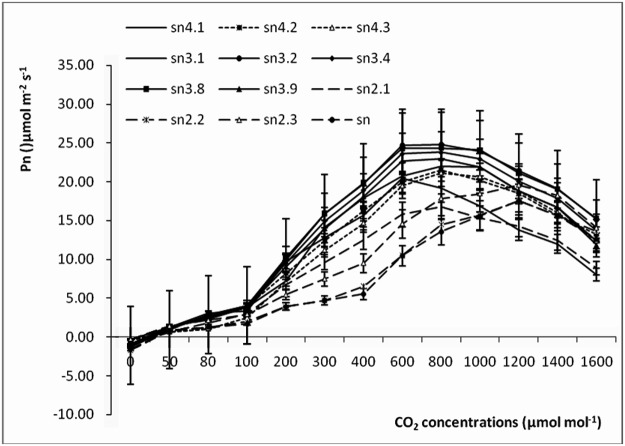
Pn-Ca curves of poplar clones of different ploidies.

**Fig 8:**
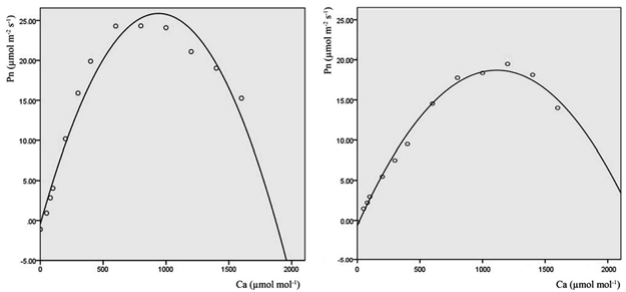
Pn-Ca simulated curve of clone sn3.8 (left) and sn2.3 (right). Solid lines are the simulated curves and white circles are the observed data.

The Pn-Ca curves of different poplar clones are shown in Fig 7. The Pn-Ca curves were shaped like an inverted “V”. As Ca increased, Pn increased initially and then decreased. The pattern was the same as the Pn-PPFD curves; triploid clones showed higher values than diploid and tetraploid clones. In particular, sn3.2 and sn3.8 showed clearly higher values at different CO_2_ concentrations. The Pn-Ca curves were also simulated using quadratic models; the simulated curves for sn3.8 and sn2.3 are shown in Fig 8 and the model parameters are presented in Table 4. The R^2^ values of all curve models were higher than 0.9, which indicated that all models were highly efficient. The diploid poplar clones showed higher average CSP and CCP values than the triploids and tetraploids. The average Pn value of the CO_2_ concentration at the CSP of diploid clones was lower than in other ploidy level poplar clones. Clone sn3.8 still showed a higher Pn at the CSP, but sn had a lower value.

**Table 4:** Coefficients and variation parameters for each Pn-Ca simulated curve.

Ploidy	Clone	a	b	c	CSP	CCP	Pn	*R* ^*2*^
Tetraploid	sn4.1	-2.573E-05	0.045	0.339	865.363	11.341	19.605	0.935
	sn4.2	-2.484E-05	0.047	-0.415	945.794	24.341	21.807	0.975
	sn4.3	-2.485E-05	0.048	-1.334	958.039	18.604	21.475	0.988
	Average				923.066	18.096	20.962	
Triploid	sn3.1	-2.876E-05	0.053	-0.600	919.055	21.177	23.689	0.971
	sn3.2	-3.031E-05	0.057	-0.591	937.974	27.083	26.079	0.967
	sn3.4	-2.950E-05	0.055	-0.565	927.981	24.179	24.836	0.969
	sn3.8	-2.980E-05	0.056	-0.446	940.023	27.024	25.884	0.965
	sn3.9	-2.912E-05	0.053	-0.623	914.070	21.667	23.709	0.973
	Average				927.821	24.226	24.839	
Diploid	Sn	-1.166E-05	0.028	-1.555	1216.240	33.422	15.694	0.971
	sn2.1	-2.030E-05	0.038	-0.530	928.700	30.049	16.979	0.980
	sn2.2	-1.174E-05	0.029	-1.599	1214.183	34.145	15.705	0.971
	sn2.3	-1.572E-05	0.035	-0.746	1114.362	8.706	18.775	0.991
	Average				1118.371	26.580	16.788	

Parameters a, b and c are coefficients of the quadratic function. *R* ^*2*^ is the criterion for evaluating the effectiveness of the model. The unit for CSP and CCP is μmol mol^-1^ and the unit for Pn is μmol m^-2^ s^-1^.

### Instantaneous photosynthetic and chlorophyll fluorescence factors

ANOVA analyses of photosynthetic and chlorophyll fluorescence factors among different clones are shown in Table 5. All factors showed significant differences (*P* < 0.01) among clones or ploidies levels. PCV varied from 2.38% (Fv/Fm) to 56.71% (Gs) and repeatability ranged from 0.745 (Ci) to 0.987 (Fv/Fm). The average values for each photosynthetic factor under conditions of leaf temperature 28°C, PPFD 1400 μmol m^-2^ s^-1^, RH 60% and Ca 400 μmol mol^-1^ are shown in Table 6. The triploids showed high Pn, Gs and Tr, but low Ci values. As with the photosynthetic factors, the triploid poplar clones had higher Fv/Fm values than the diploid and tetraploid clones.

**Table 5:** ANOVA analyses for *PCV* and *R* of clones of different ploidies and clones in photosynthetic and chlorophyll fluorescence traits.

Traits	Variation source	*SS*	*df*	*MS*	*F*	*P* value	*PCV* (%)	*R*
Pn	Ploidies	197.450	2	98.725	30.582	0.000**		
	Clones	279.979	11	25.453	25.453	0.000**	16.29	0.961
Gs	Ploidies	2.472	2	1.236	26.497	0.000**		
	Clones	3.899	11	0.354	76.298	0.000**	56.71	0.987
Ci	Ploidies	9426.351	2	4713.176	6.113	0.006**		
	Clones	14620.579	11	1329.144	3.922	0.002**	10.68	0.745
Tr	Ploidies	78.277	2	39.138	33.372	0.000**		
	Clones	108.312	11	9.847	27.266	0.000**	31.77	0.963
Fv/Fm	Ploidies	0.010	2	0.005	62.893	0.000**		
	Clones	0.013	11	0.001	79.311	0.000**	2.38	0.987

** indicates that the variance is significant at the 0.01 level.

**Table 6:** Average photosynthetic and chlorophyll fluorescence traits of hybrid clones of different ploidies.

Ploidy	clone	Pn	Gs	Ci	Tr	Fv/Fm
Tetraploid	4.1	15.90	0.40	311.00	5.19	0.813
	4.2	16.30	0.63	300.00	5.41	0.815
	4.3	18.00	0.53	310.00	6.23	0.800
	Average	16.73	0.52	307.00	5.61	0.809
Triploid	3.1	20.33	1.21	276.00	8.63	0.828
	3.2	22.50	0.93	274.67	7.65	0.838
	3.4	18.20	0.58	293.67	5.84	0.815
	3.8	23.80	1.21	259.67	8.63	0.846
	3.9	19.30	0.52	300.00	5.86	0.827
	Average	20.83	0.89	280.80	7.32	0.831
Diploid	2.1	16.80	0.37	307.00	4.82	0.792
	2.2	14.80	0.26	321.00	3.27	0.775
	2.3	14.63	0.22	324.00	3.39	0.786
	2.4	16.57	0.30	316.00	4.13	0.798
	Average	15.70	0.29	317.00	3.90	0.788

The units for Pn, Gs, Ci and Tr are μmol m^-2^ s^-1^, μmol m^-2^ s^-1^, μmol mol^-1^ and mol m^-2^ s^-1^, respectively.

### Correlation coefficients

The correlation coefficients of different traits are shown in Table 7. All pair-wise correlation coefficients were significant (*P* < 0.01). The correlation coefficients among growth traits (H, BD, LN, LA, LFW, SFW, LDW and SDW) ranged from 0.512 to 0.857. There were also significant positive correlations between Pn and Gs, Pn and Tr, and Gs and Tr, but Ci was negatively correlated with the other photosynthetic factors. All growth traits were positively correlated with Pn, Gs and Tr, and negatively correlated with Ci. Fv/Fm was positively correlated with growth traits. As with growth factors, Fv/Fm was positively correlated with Pn, Gs and Tr, and negatively correlated with Ci.

**Table 7:** Pair-wise correlation coefficients of different traits.

Trait	BD	LN	LA	SFW	SDW	LFW	LDW	Pn	Gs	Ci	Tr	Fv/Fm
H	0.657**	0.692**	0.786**	0.840**	0.657**	0.726**	0.638**	0.755**	0.756**	-0.489**	0.748**	0.858**
BD		0.535**	0.567**	0.681**	0.512**	0.618**	0.584**	0.640**	0.673**	-0.460**	0.674**	0.748**
LN			0.690**	0.716**	0.678**	0.536**	0.655**	0.705**	0.727**	-0.518**	0.663**	0.758**
LA				0.769**	0.725**	0.717**	0.632**	0.794**	0.710**	-0.575**	0.739**	0.820**
SFW					0.857**	0.764**	0.646**	0.842**	0.810**	-0.598**	0.822**	0.887**
SDW						0.689**	0.594**	0.768**	0.803**	-0.573**	0.722**	0.770**
LFW							0.586**	0.658**	0.626**	-0.557**	0.607**	0.739**
LDW								0.533**	0.633**	-0.502**	0.593**	0.633**
Pn									0.858**	-0.449**	0.920**	0.856**
Gs										-0.469**	0.938**	0.839**
Ci											-0.457**	-0.660**
Tr												0.845**

**Correlation is significant at the 0.01 level (2-tailed).

## Discussion

Polyploidy is an important force in plant evolution [31]. In general, because of the increased heterozygosity and genic and biochemical flexibility provided by the additional alleles, polyploid plants are often larger and have larger cells. Polyploids are considered better able to colonize a wider range of habitats and survive in harsh, unstable environments [23], [31]. The major cytological mechanisms of polyploidy are the union of unreduced gametes, somatic doubling and polyspermy [32]. The union of unreduced gametes used in this research is one of the most important mechanisms for obtaining polyploids [32]. In terms of growth traits at different ploidies, Zhu [24] found that there were significant differences between diploid and tetraploid *P*. *davidiana* Dode hybrid clones in terms of tree height, basal diameter and chlorophyll content. In 1995, Zhu found that triploid *P*. *tomentosa* clones grew faster than diploid clones [33]. In this study, triploid poplar clones had clearly higher values for all growth traits compared with diploid and tetraploid poplar clones. Tetraploid poplar clones did not show superiority to diploid poplar clones in growth traits, which agreed with the results of Comai [34] who indicated that because of the ploidy increase, increased numbers of chromosomes led to increased numbers of cells and nuclei, thereby increasing the possibility of producing aneuploidy in meiosis and mitosis, resulting in epigenetic instability.

The most common parameter used to describe the extent of variability in a breeding population is the average PCV. In this study, the average PCVs of the traits ranged from 2.38% (Fv/Fm) to 56.71% (Gs). Except for Fv/Fm, the PCV of all traits exceeded 10%. The repeatability value indicates the reliability with which a genotype can be recognized by its phenotypic expression. In this study, the estimates of repeatability for all traits at the clone mean level ranged from 0.656 (LFW) to 0.987 (Fv/Fm), which is in general agreement with research by Kien [35]. The high PCV and high repeatability indicated that the variation of each trait in poplar hybrid clones with different ploidies is not significantly influenced by environmental factors and that selection and evaluation of these clones will be effective [36].

Light can affect the enzyme activity and stomatal aperture of plant leaves. Without light, there is no photosynthesis. The light compensation point is a critical value; at this point, the amounts of CO_2_ used in photosynthesis and created by respiration are equal. The light saturation point is a limiting value; over this value, the photosynthetic rate does not increase, and may decrease [37]. The LSP in plants is important under high light conditions, as the dark reaction follows the light reaction in photosynthesis and then limits the instantaneous photosynthetic rate [38]. Poplars are shade-avoiding species, and our results showed that they had a high photosynthetic rate (15.90–25.01 μmol m^-2^ s^-1^) when the light radiation reached the LSP. The five triploid clones had higher LSPs and lower LCPs, indicating that triploid clones can adapt to extensive light illumination intensities. These results were similar to those of Fu [39] for rice and Qi [16] for poplar. At the LSP, the Pn values of the triploid clones were higher than those of the tetraploid and diploid clones. There were no significant variations between the tetraploid and diploid clones. These variations might reflect differences in leaf structure, composition, transcriptional level, methylation of DNA or nucleolus dominance in clones of different ploidies, which should be explored in future research [16]. As the light illumination intensity increased, Gs and Tr showed the same regular pattern as Pn, indicating that the photosynthetic indices interacted with each other during photosynthesis. These results are in agreement with research carried out on *P*. *tomentosa* [26] and *Catalpa bungei* [27].

CO_2_ is the most important substrate in photosynthesis. Within a certain concentration range, enhanced CO_2_ concentration can promote the instantaneous photosynthetic rate [40]. There is strong evidence that plants have already responded to the increase in atmospheric CO_2_ concentration [41]. Atmospheric CO_2_ concentrations are projected to double from the current concentration of 350 μmol mol^-1^ to 700 μmol mol^-1^, which will further stimulate plant growth and ecosystem changes within the next 80 years [37]. CO_2_ concentration is the main limiting factor of photosynthesis for plants when the temperature and humidity are moderate and the light radiation is saturated. In this study, as the CO_2_ concentration increased, the Pn values of poplar clones with different ploidies initially increased and then decreased. The CSP values of the diploids were obviously higher than those of the tetraploid and triploid poplar clones. However, at the CSP, the Pn values of the triploids were higher than those of the diploids and tetraploids. These results suggested that after 80 years, when the CO_2_ concentration is higher than 700 μmol mol^-1^, diploid poplar may be more preferable than triploid and tetraploid because of its higher CSP.

Chlorophyll fluorescence is a measure of photosynthetic performance and is widely used by plant physiologists and ecophysiologists [42]. The ratio Fv/Fm is the most frequently used parameter in ecophysiological research to determine the maximum photochemical efficiency of PSII [43, 44]. This ratio, typically ranging between 0.75 and 0.85, is proportional to the effectiveness of light energy utilization under standard conditions of CO_2_ fixation and to the quantum yield of photochemical processes [45]. In this study, Fv/Fm values ranged from 0.775 to 0.846, which indicated that the plants were not under stress and that the greenhouse conditions were appropriate for poplar growth. However, the Fv/Fm values were higher in the triploid and tetraploid clones than in the diploid clones. Polyploid poplar clones might have higher pre-resistance than diploid poplar clones [46].

Ceulemans [47] and Ma [48] indicated that the Pn and Gs of *Populus* clones were positively correlated with growth traits; Pn and Gs are tightly coupled [49]. In this research, strong positive correlations between growth traits and Pn, Gs, Tr and Fv/Fm were observed, and there were also significant correlations among all photosynthetic parameters, indicating that all traits influence and restrict each other, ultimately controlling plant growth.

## Conclusion

In conclusion, there were significant variations in growth, photosynthetic and chlorophyll fluorescence (Fv/Fm) traits between different clones with ploidies, and there were also significant correlations between different traits, which suggested that all traits collaborated to promote plant growth. Triploid clones showed higher growth traits and higher instantaneous photosynthetic and chlorophyll fluorescence factors than diploid and tetraploid clones, which indicated that triploid clones were preferable at the seedling stage. Clone sn3.8 showed high growth traits, Pn and Fv/Fm values, and should be investigated further in future studies. Additional investigations are also needed to determine the transcriptomic, proteomic, metabolomic and structural differences between poplars with different ploidies, to explain the variation in chromosome polyploidy, and to improve the genetic manipulation of poplar.
